# Supplementary material for: Identifying microbiota community patterns important for plant protection using synthetic communities and machine learning
Source: Nat Commun. 2023 Dec 2;14:7983. doi: 10.1038/s41467-023-43793-z (PMC10693592; doi:10.1038/s41467-023-43793-z)
Supplement: Supplementary file 1 — Supplementary Information [file 41467_2023_43793_MOESM1_ESM.pdf]

Identifying microbiota community patterns important for plant protection using synthetic communities and machine learning

Barbara Emmenegger[1], Julien Massoni*[1], Christine M. Pestalozzi[1], Miriam Bortfeld-Miller, Benjamin A. Maier, Julia A. Vorholt*

Institute of Microbiology, ETH Zurich, Zurich, Switzerland

* correspondence to: julien.massoni@biol.ethz.ch; jvorholt@ethz.ch

Supplementary Figures 1 - 7

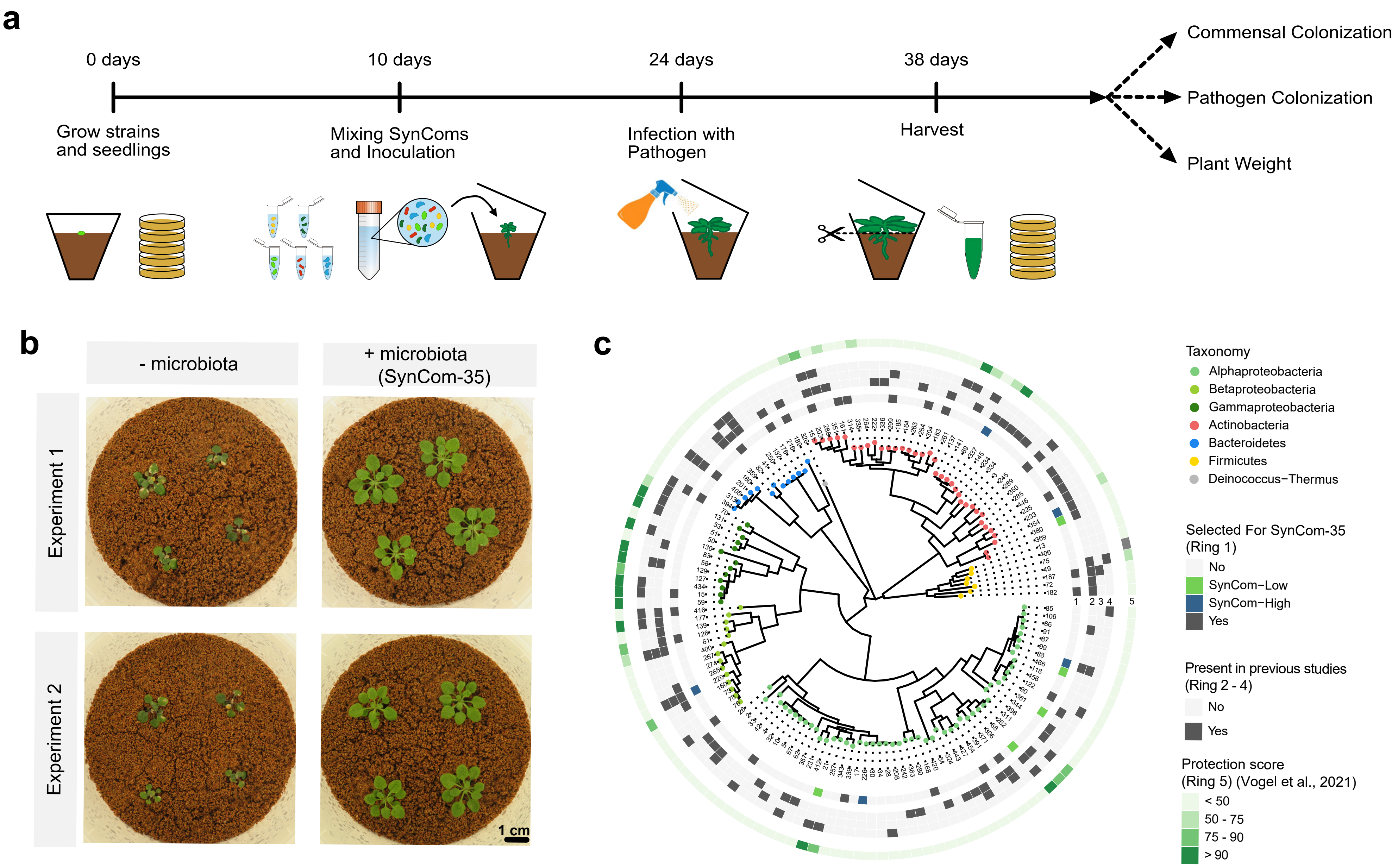

Figure S1: Experimental design, host traits of interest, and bacteria collection. a. Experimental procedure for plant treatments. b. Illustration of the influence of the phyllosphere microbiota on plant phenotype. c. Phylogenetic tree of the SynCom-137 (79) including single ASVs of the *At*-LSPHERE (omitting Leaf in front of number) with information of strain selection in surrounding rings. Ring 1 shows whether a strain was included in this study. Rings 2 to 4 illustrate whether a strain was included in previous studies that used *At*-LSPHERE strains, in order Carlström et al. (55), Schäfer et al. (76), and Maier et al. (75). Ring 5 illustrates plant protection as analyzed in a previous study (45).

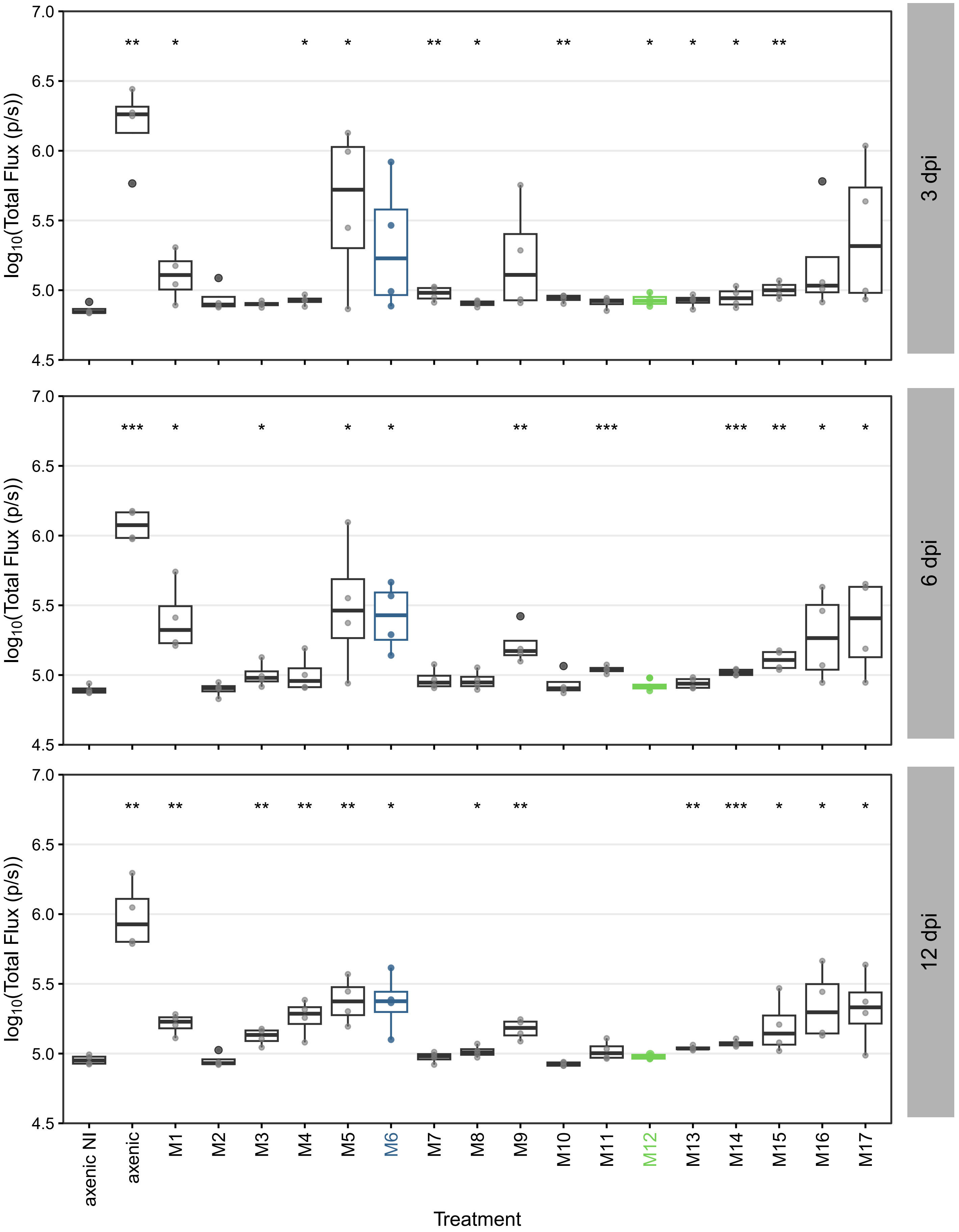

Figure S2: Pathogen luminescence measurements in the pilot experiment for the two controls axenic non-infected (axenic NI) and axenic infected (axenic), and the 17 randomly assembled Mini5SynCom (M1 to M17) (n = 4). Each data point corresponds to the median of the luminescence of four plants in a microbox. Significance levels for mean comparisons between the axenic non-infected control and all other treatments were obtained with one-sided Welsh's tests (see Table S1). M6 and M12 were included in the Mini5SynCom screen (SynCom-Low and SynCom-High, respectively), and are coloured accordingly. Abbreviations: dpi, days post infection. Significance code: NS > 0.05; * ≤ 0.5; ** ≤ 0.01: *** ≤ 0.001; **** ≤ 0.0001.

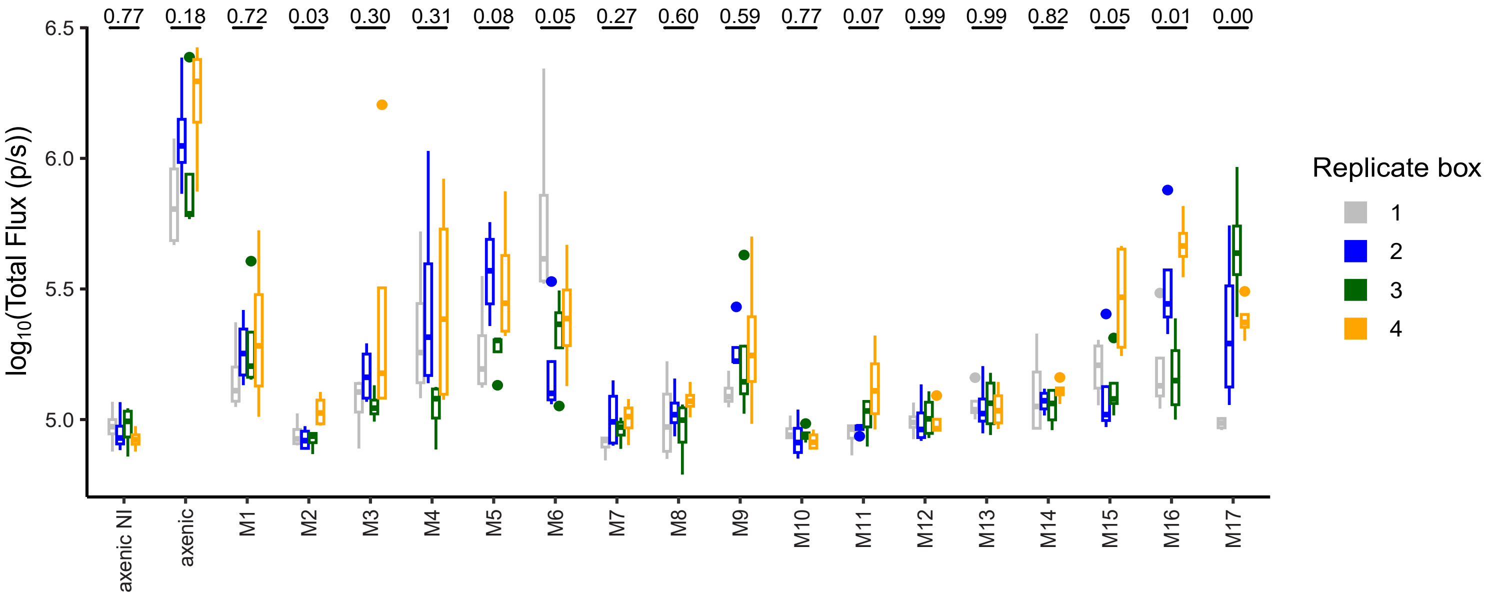

Figure S3: Boxplot of the log_{10}-transformed luminescence presented for each Mini5SynCom and the axenic infected (axenic) and axenic non-infected (axenic NI) controls of the pilot experiment (n = 4 per boxplot). *P-values* are presented at the top of each treatment and were calculated with ANOVAs with replicate box as independent variable, without multiple testing *p-value* correction. The boxplots show median with hinges corresponding to first and third quartiles, and whiskers extending to 1.5 times the inter quartile range.

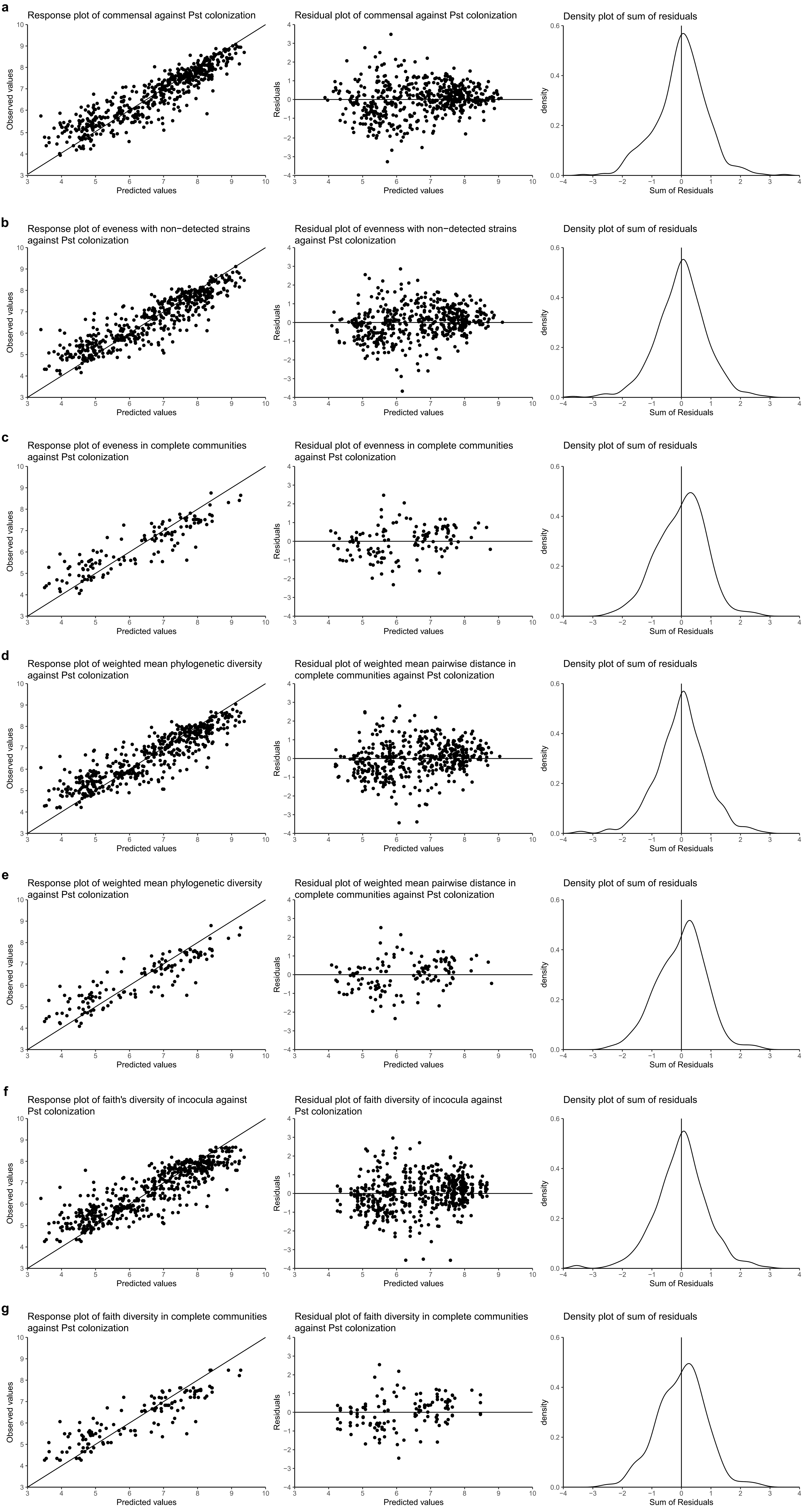

Figure S4: Diagnostic plots of the best linear mixed models for regression analyses with pathogen colonization as dependent variable, and experiment and box random intercepts and slopes for the box as random effects. a. Overall Mini5SynCom commensal colonization as fixed effect. b-c. Evenness of Mini5SynComs as fixed effect. b. For communities with no ambiguous abundances. c. For communities with no ambiguous abundances and no strain below level of detection. d-e. Weighted mean pairwise distances of Mini5SynComs as fixed effect. d. For communities with no ambiguous abundances. e. For communities with no ambiguous abundances and no strain below level of detection. f-g. Faith's phylogenetic distance as fixed effect.
f. For all communities (calculation on inoculum composition). g. For communities with no ambiguous abundances and no strain below level of detection.

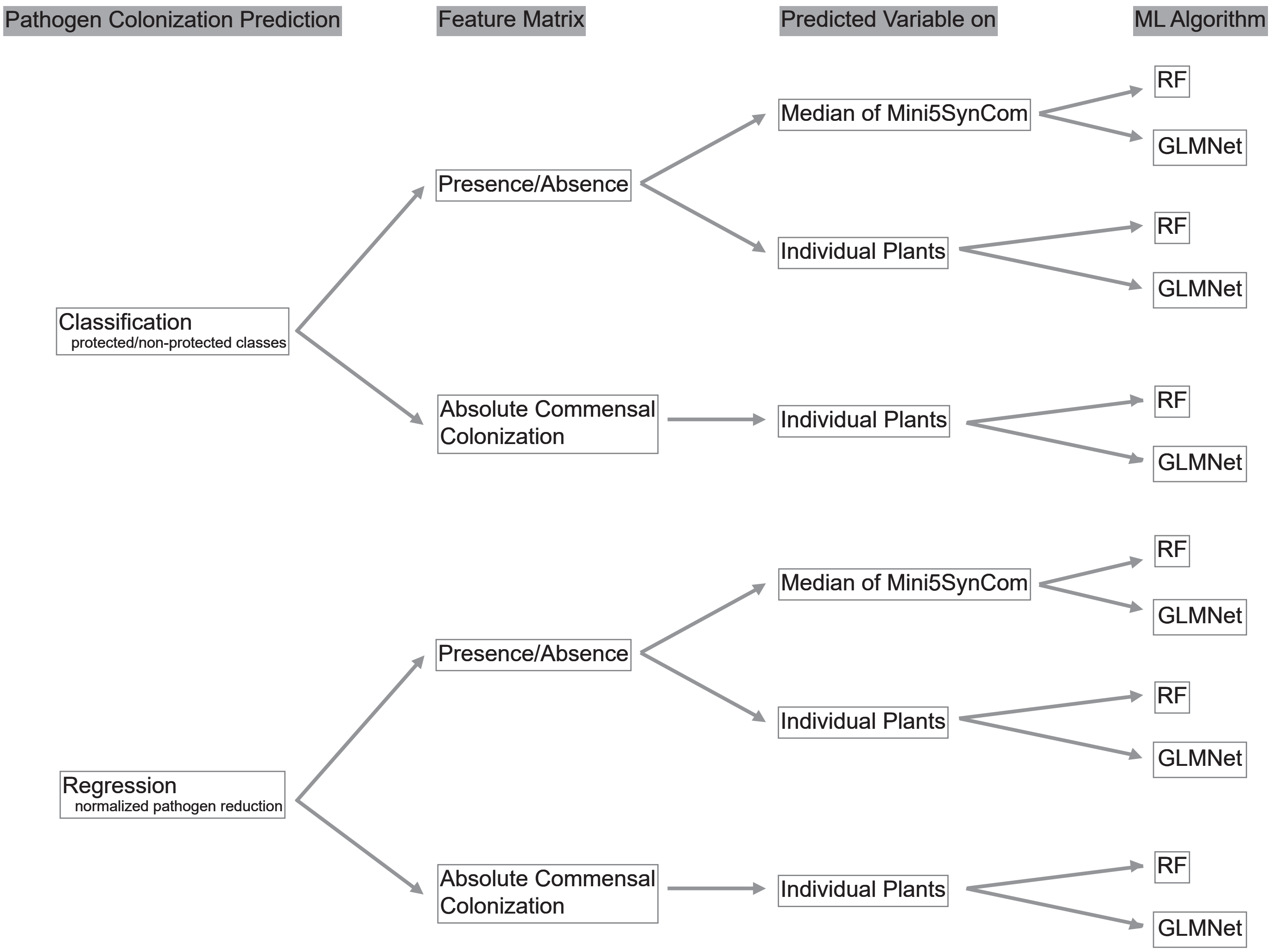

Figure S5: Diagram of the different algorithms used in machine learning. First level is the predicted variable (class or value). Second level is the type of predictive variables used (presence/absence or absolute colonization of Mini5SynCom commensals). Third level is the type of measurements used to define the predicted variable (median of Mini5SynCom or individual plant samples). Last level is the method used (GLMNet or RF).

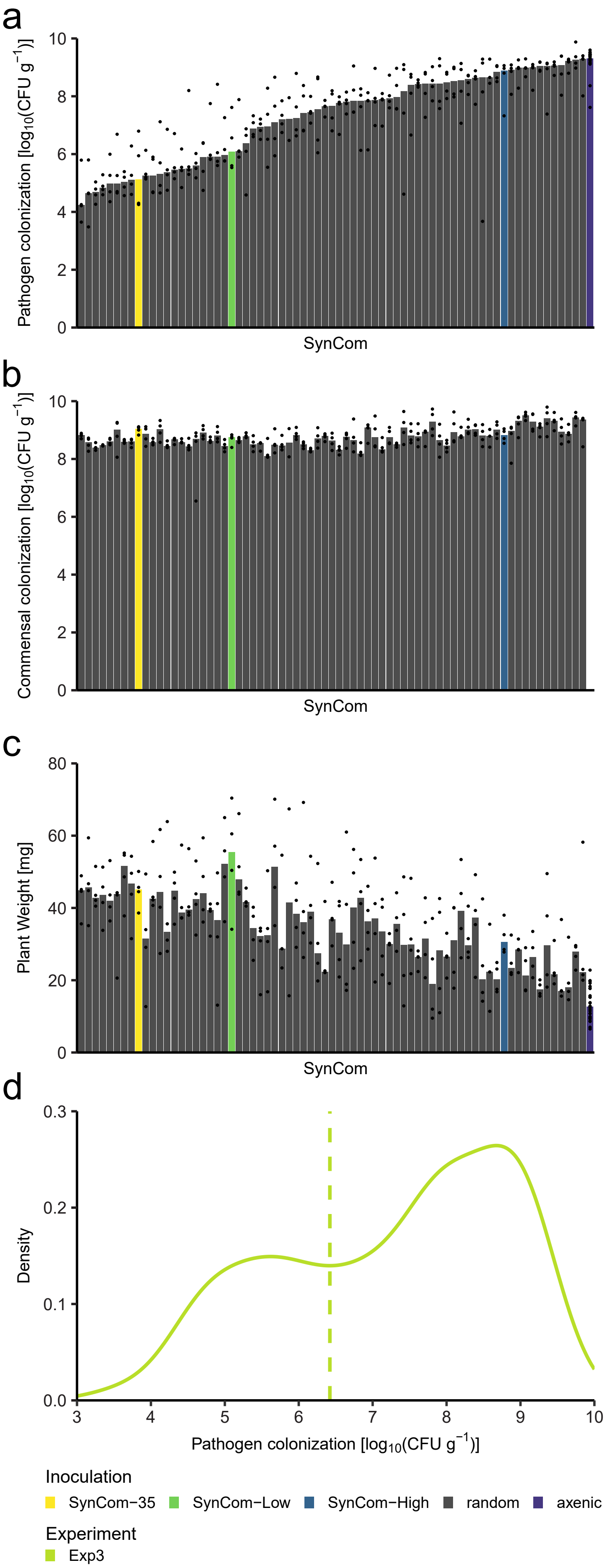

Figure S6: Strain colonization and plant weight in the test dataset (experiment 3). A-C. Each bar represents the median for each treatment; points are individual-plant measurements (n = 4); x-axes represent individual treatments (n = 72) following the same sorting in all panels. A. Pathogen colonization. B. Overall Mini5Syncom commensal colonization. C. Plant weight. D. Density curve of the pathogen colonization. Abbreviation: Exp3, experiment 3.

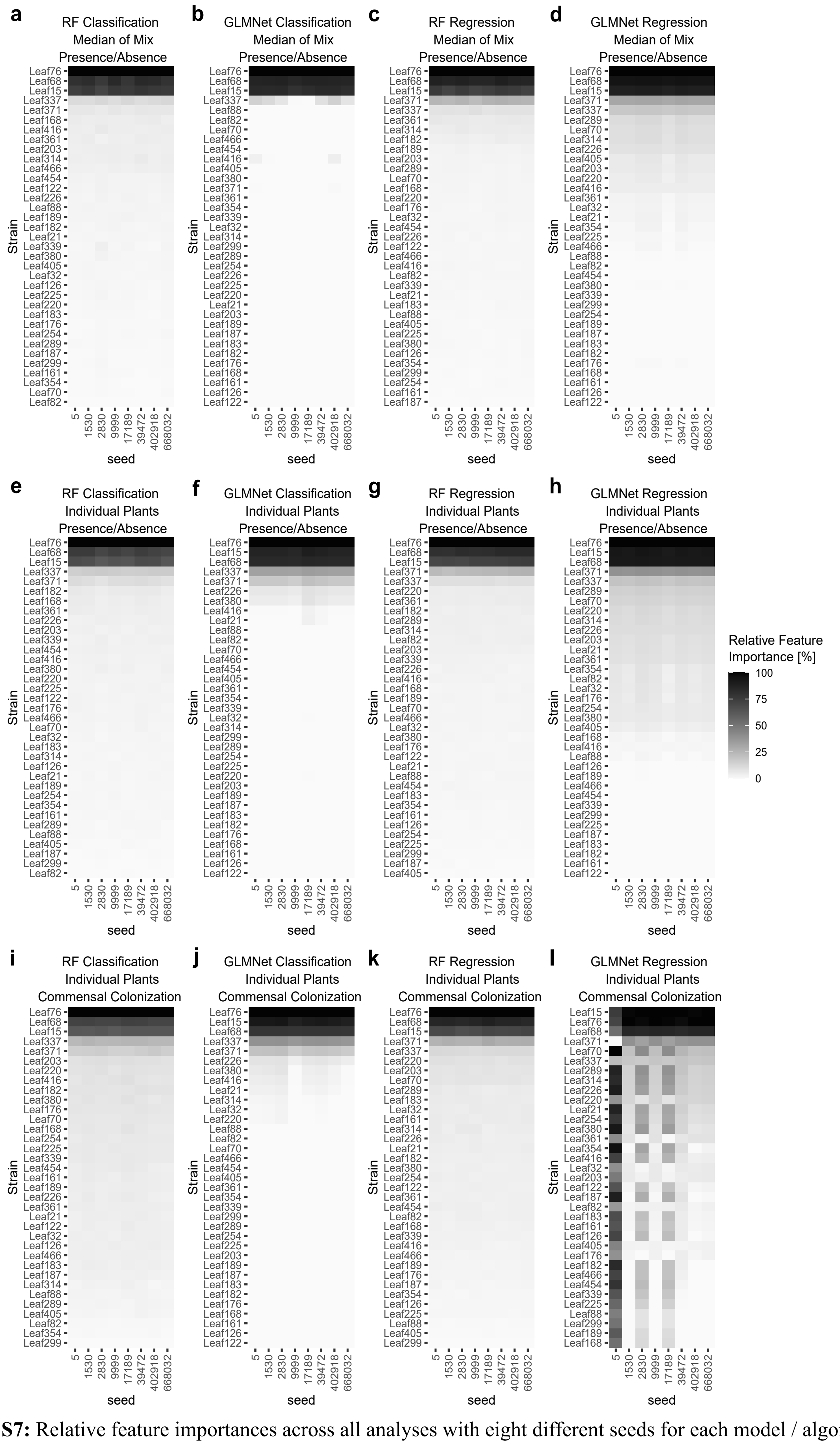

Figure S7: Relative feature importances across all analyses with eight different seeds for each model / algorithm combination. Above each plot the algorithm, the commensal measurement, and the type of predicted variables are indicated. The strains are ordered according to the median of their relative importance across the eight seeds for each model / algorithm combination. Abbreviations: RF, random forest; GLMNet, elastic-net regularized generalized linear models.
