# Supplementary material for: Identifying microbiota community patterns important for plant protection using synthetic communities and machine learning
Source: Nat Commun. 2023 Dec 2;14:7983. doi: 10.1038/s41467-023-43793-z (PMC10693592; doi:10.1038/s41467-023-43793-z)
Supplement: Supplementary file 3 — Description of Additional Supplementary Files [file 41467_2023_43793_MOESM3_ESM.pdf]

Description of Additional Supplementary Files

Supplementary Data 1: One sided Welsh t-tests and Shapiro-Wilk tests of random communities in pilot experiment conducted using medians of each Mix calculated from log10-transformed pathogen luminescence.

Supplementary Data 2: SynCom-35 strain collection used in the screening experiment with taxonomical and a priori knowledge information.

Supplementary Data 3: Best linear mixed models with the log10-transformed pathogen colonization as dependent variable, and either the log10-transformed commensal colonization (centred), evenness or phylogenetic diversity as fixed effects. Best models are those with a delta AIC (Akaike information criterion) below four. Most complete models included box and experimental random effects with the first nested in the second. Measurement units and transformations of variables are indicated between squared brackets. Null hypotheses regarding coefficient values were tested with two-sided t-test procedures. Abbreviations: AIC, Akaike information criterion; LogCFUgFW, log10-transformed colonization in colony forming units per gram of plant fresh weight.

Supplementary Data 4: Percentage of bootstrapped datasets with a detected minimum used to classify samples between pathogen protected and non-protected. Data were bootstrapped 1,000 times.

Supplementary Data 5: Pathogen colonization [log10(CFU g^{-1} fresh weight)] corresponding to the local minima in density curves of the different experiments. Measurements were either individual plant measurements or medians of plant measurements for each box.

Supplementary Data 6: Performances of the classification analyses. The results are the medians calculated across the eight seeds used to initiate pseudo-random processes. Abbreviations: ML, machine learning; GLMNet, elastic-net regularized generalized linear models; RF, random forest; NA, not applicable.

Supplementary Data 7: Performances of the regression analyses. The results are the medians calculated across the eight seeds used to initiate pseudo-random processes. The global-average algorithm consists in predicting the pathogen colonization to be equal to the average colonization of the entire dataset. Abbreviations: RMSE, root-mean-squared error; ML, machine learning; GLMNet, elastic-net regularized generalized linear models; RF, random forest; NA, not applicable.

Supplementary Data 8: Relative importances of strains in all machine learning analyses. The results are the medians calculated across the eight seeds used for each model-method combination. Abbreviations: ML, machine learning; GLMNet, elastic-net regularized generalized linear models; RF, random forest.

Supplementary Data 9: Relative importances of strains in random forest when varying the global minima used to define the classes in the training dataset (experiments 1 and 2). The dependent variable is the pathogen colonization measured on individual plants, and the independent variables are presence/absence of strains in Mini5SynComs. The local minima consisted in 10 random sampling of the bootstrapped distributions of global minima in experiments 1 and 2. Abbreviation: Rep, replicate.

Supplementary Data 10: Model selection with Akaike information criterion for regression analyses of the validation experiment of machine-learning results. Pathogen colonization was the dependent variable, strain inoculation with pathogen-reducing and random-strains was a fixed effect, and the replicates of the experiment was a random effect. Each data point is the average of pathogen colonization for the four plants of one box. Abbreviations: AIC, Akaike information criterion.

Supplementary Data 11: Contrasts of the treatments presented in Fig. 7A. P-values were calculated with two-sided t-test procedures. The significance-group lettering was based on a 0.05 significance level after Bonferroni correction calculated from the family of all pairwise comparisons of treatments presented in this panel of the Fig. 7. Abbreviation: Bonf., Bonferroni.

Supplementary Data 12: Contrasts of the treatments presented in Fig. 7B. P-values were calculated with two-sided t-test procedures. The significance-group lettering was based on a 0.05 significance level after Bonferroni correction calculated from the family of all pairwise comparisons of treatments presented in this panel of the Fig. 7. Abbreviation: Bonf., Bonferroni.

Supplementary Data 13: Model selection with Akaike information criterion for regression analyses of the validation of combination of strains reducing pathogen colonization. Pathogen colonization was the dependent variable, strain inoculations was a fixed effect, and the replicates of the experiment was the random effect. Each data point is the average of pathogen colonization for the four plants of one box. Abbreviations: AIC, Akaike information criterion.

Supplementary Data 14: Results of the regression analyses to validate the additive effect of Leaf371 and Leaf337 on pathogen reduction for the two best models with delta AIC inferior to four. Two-sided t-test procedures were conducted to reject null hypotheses regarding coefficients of the models.

Supplementary Data 15: Contrasts of the treatments presented in Fig. 8D. P-values were calculated with two-sided t-test procedures. The significance-group lettering was based on a 0.05 significance level after Bonferroni

correction calculated from the family of all pairwise comparisons of treatments presented in the fig. (with the exclusion of SynCom35). Abbreviation: Bonf., Bonferroni.
